# Quantitative LC-MS/MS analysis of seven ginsenosides and three *aconitum* alkaloids in Shen-Fu decoction

**DOI:** 10.1186/1752-153X-7-165

**Published:** 2013-10-10

**Authors:** Na Guo, Mingtao Liu, Dawei Yang, Ying Huang, Xiaohong Niu, Ruifan Wu, Ying Liu, Guizhi Ma, Deqiang Dou

**Affiliations:** 1 Experimental Research Center, China Academy of Chinese Medical Sciences, Beijing 100700, China; 2 SRI International, Menlo Park, CA 94025, USA; 3 Key Laboratory of Biofuels, Qingdao Institute of Bioenergy and Bioprocess Technology, Chinese Academy of Sciences, Songling road 189, Qingdao 266101, China; 4 College of Pharmacy, Xinjiang Medical University, Urumqi 830011, China; 5 Key Laboratory of Bioactive Substances and Resource Utilization of Chinese Herbal Medicine, Ministry of Education, Institute of Materia Medica, Chinese Academy of Medical Sciences and Peking Union Medical College, Beijing 100050, China; 6 Department of Chinese Medicine Chemistry, Liaoning University of Traditional Chinese Medicine, Dalian 116600, China

**Keywords:** Ginsenosides, Aconitum alkaloids, Shen-Fu decoction, RRLC-MS/MS

## Abstract

**Background:**

Shen-Fu decoction is a traditional Chinese medicine prescription with a 3:2 ratio of *Radix Ginseng* and Fuzi **(*Radix Aconiti lateralis praeparata*)**. Ginsenosides and alkaloids are considered to be the main active components of Shen-Fu decoction. However, no analytical methods have been used to quantitatively analyse both components in Shen-Fu decoction simultaneously.

**Results:**

We successfully developed a rapid resolution liquid chromatography coupled with tandem mass spectrometry (RRLC-MS/MS) method for the simultaneous analysis of seven ginsenosides and three aconitum alkaloids in Shen-Fu decoction, the decoction of Radix ginseng and Fuzi **(*Radix Aconiti lateralis praeparata*)**. Chromatogrpahic separation by RPLC was achieved using a reversed-phase column and a water/acetonitrile mobile phase, containing 0.05% formic acid and using a gradient system. The method was optimized to allow for simultaneous analysis of all analytes in 11minutes without the need for baseline resolution of the components. Furthermore, the separation demonstrated good linearity (r > 0.9882), repeatability (RSD < 7.01%), intra- and inter-day precisions (RSD < 5.06%) and high yields of recovery (91.13-111.97%) for ten major constituents, namely ginsenoside-Re, Rg_1_, Rb_1_, Rc, Rb_2_, Rd, Rf, aconitine, hypacoitine and mesaconitine.

**Conclusions:**

The developed method could be used as a rapid and reliable approach for assessment of the quantity of the major constituents in Shen-Fu decoction.

## Background

**Figure 1:**
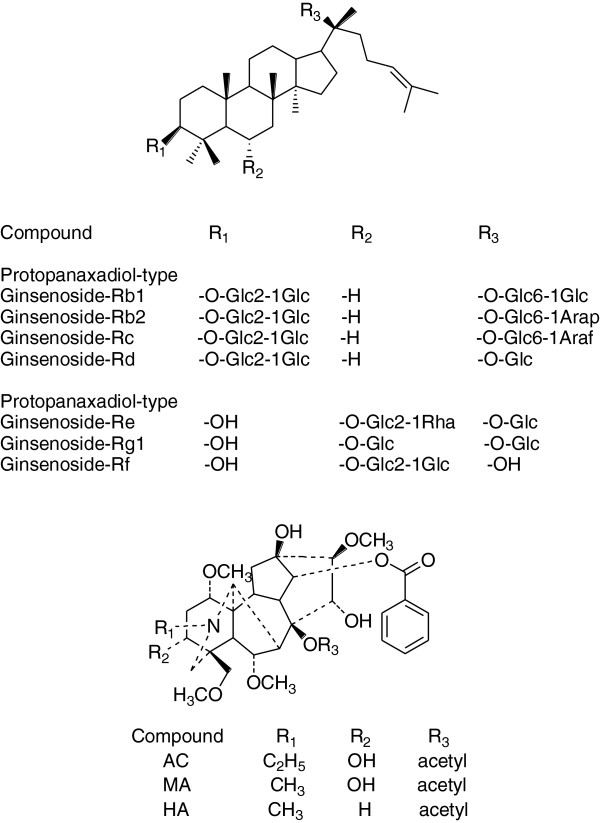
Chemical structures of ginsenosides and Aconitum alkaloids analyzed in Shen-Fu decoction.

Decoction is the traditional prescription of traditional Chinese medicines (TCMs). Based on TCM theory, one single herb or several kinds of herbs combined are boiled in water to make the decoction. First documented in 1465, Shen-Fu decoction is a TCM prescription with a 3:2 ratio of *Radix Ginseng* and Fuzi **(*Radix Aconiti lateralis praeparata*)**. Both components have been commonly used as herbal medicines in China for about 1800 years, predominantly used for folk treatment of diseases with the sign of Yangqi decline or Yang exhaustion. Shen-Fu decoction is also used to treat cardiovascular diseases such as circulatory collapse, shock, thoracic obstruction and acute thoracic pain. Shen-Fu Injection (SFI for intravenous medication), is a typical form of Shen-Fu decoction, that has been used for treatment of many kinds of diseases because of its cardiovascular protective effectiveness [1-3]. The main active components found in Shen-Fu decoction are ginsenosides and alkaloids. Ginsenosides are generally classified into four groups: protopanaxadiol, protopanaxatriol, ocotillol and oleanolic acid type [4-6], Currently, more than 150 ginsenosides have been isolated and identified in the literature. Among them, ginsenosides-Rb_1_, Rb_2_, Rc, Rd, Rg_1_, Re and Rf (Figure 1) are the most important compounds in chemical analysis of ginsengs. At present, about 224 alkaloids have been isolated and identified from Aconitum [7,8]. These have been classified into four major groups, nonester alkaloids (NEAs), monoester diterpene alkaloids (MDAs), diester diterpene alkaloids (DDAs) and lipoalkaloids. *Aconitum* alkaloids are mainly constituted of three DDAs, diester-diterpence called aconitine (AC), measaconitine (MA) and hypaconitine (HA) (Figure 1). They are known for their high toxicity and pharmacological activity, as well as being the target markers of Fuzi. In general, the curative effect of traditional Chinese medicine is an integrative result of a number of ginsenosides and alkaloids. In order to minimize the variability of active ingredients in the decoction and ensure repeatable and reproducible therapeutic effects, it is very important to establish quality control methodology for the decoction. To this end, analysis of ginsenosides and *Aconitum* alkaloids is required to assess the quality of Shen-Fu decoction.

Previous methods that have been used to analyze ginsenosides and alkaloids include HPLC-DAD (ELSD), CE, GC-MS and LC-MS [9-19] and alkaloids [20-30]. In comparison with traditional HPLC, RRLC provides a higher peak capacity, greater resolution, increased sensitivity and higher speed of analysis. When coupled to a triple quadrupole tandem mass spectrometer (QQQ-MS/MS), it can achieve high sensitivity and selectivity by using the multiple reaction monitoring (MRM) scan mode without the baseline chromatographic separation of target analytes. This method greatly facilitates the quantification of chemical markers in complex matrixes with only a small amount of sample. To date, there are no studies reporting the simultaneously quantitative determination of ginsenosides and Aconitum alkaloids in Shen-Fu decoction. The primary aim of the present study was to develop a direct and rapid RRLC-MS/MS method for simultaneously quantifying the ten constituents in Shen-Fu decoction, namely, ginsenosides-Rb1, Rb2, Rc, Rd, Rg1, Re and Rf and Aconitum alkaloids including AC, MA and HA.

## Results and discussion

### Chromatographic conditions and MS/MS method development

**Figure 2:**
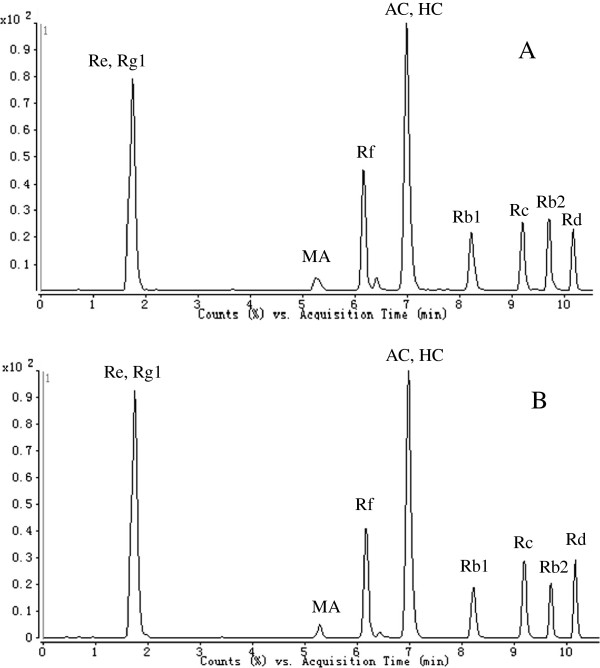
Typical RRLC-QQQ MS/MS chromatograms of marker chemicals in Shen-Fu decoction (A) standard mixture (B) Shen-Fu decoction.

Different mobile phases, including acetonitrile with 0.05%, 0.1% aqueous formic acid, acetic acid, 5 mM and 10 mM ammonium formate solutions were tested. The best peak shape and resolution was obtained with a mixture of acetonitrile and aqueous 0.05% formic acid solution. Using an optimized elution gradient, the main components were separately eluted within 11 min. The typical RRLC-QQQ MS/MS chromatograms of the marker chemicals in Shen-Fu decoction are shown in Figure 2. In order to increase sensitivity and specificity of quantification, multiple reaction monitoring was performed. All factors related with MS performance including ionization mode, capillary voltage, fragmentor voltage, collision energy, gas flow and desolvation temperature were analyzed. The optimum conditions were determined as follows: postive ion mode, capillary voltage 4000 V, drying gas, gas temperature 350°C and nebulizer pressure of 50 psi.

Optimization of this MS/MS method produced highest achiveable response using the MRM pairs comprising of the precursor and product ions, which can achieve better quantitation than reported results using the selected ion monitoring (SIM) mode. After optimization, the precursor and product ions of the ten analytes were recorded (Table 1). The optimum collision energy was determined to be 50 eV for Ginsenoside Re, 40 eV for Rg_1_, 55 eV for Rf and Rd, 65 eV for Rb_1_, Rc and Rb_2_. For alkaloids, they required a lower collision energy of 35 eV for MA, 40 eV for HA and 45 eV for AC (Table 1).

**Table 1:** Mass spectra properties of ten compounds in Shen-Fu decoction

**Compound name**	**Precursor ion**	**Product ion**	**Frag(V)**	**CE(V)**
Ginsenoside Re	969.6	789.5	150	50
Ginsenoside Rg_1_	823.5	643.5	135	40
Ginsenoside Rf	823.3	365.3	140	55
Ginsenoside Rb_1_	1131.6	365.0	150	65
Ginsenoside Rc	1101.7	335.0	150	65
Ginsenoside Rb_2_	1101.6	334.8	150	65
Ginsenoside Rd	969.9	789.3	150	55
Aconitine	646.4	586.4	135	45
Mesaconitine	632.3	572.3	135	35
Hypacoitine	616.3	556.2	135	40

### Method validation

To determine the reliability of the test results, the method validation included linearity, repeatability, intra- and inter-day precisions and recovery test. The standard calibration curves of all compounds were shown in Table 2 with satisfactory linearity (r > 0.9882). *Aconitum* alkaloids had a linear range of 0.03 ng mL^-1^ to 6.24 ng mL^-1^, whereas ginsenosides displayed a wider linear range of 3.90 ng mL^-1^ to 125.00 ng mL^-1^ (Table 2). The limit of dectection (LOD) ranged from 0.01 ng mL^-1^ to 1.25 ng mL^-1^ for all ten analytes. The intra-day and inter-day with RSD less than 5.06% are demonstrated in Table 2. The repeatability was satisfactory with RSD below 7.01%. Recovery of the ten compounds (Table 3) was within the range of 91.13-111.97% and showed no relevant difference in the percent yield recovered using with different concentrations of the compounds. Thus, the ten analytes can be quantitatively analyzed simultaneously in a relatively short-time using this optimized method.

**Table 2:** **Calibration curves**, **LOD**, **LOQ**, **Precision and Repeatability for ten compounds in Shen-Fu decoction**

**Compound name**	**Calibration curve**	**r**	**Linear range(ng·mL**^**-1**^**)**	**LOD(ng·mL**^**-1**^**)**	**LOQ(ng·mL**^**-1**^**)**	**Intra-day(n=6)**	**Inter-day(n=6)**	**Repeatability(n=5)**
Ginsenoside-Rb_1_	Y=11.04X+181.44	0.9930	3.90~125.00	0.97	3.00	3.44	4.11	6.51
Ginsenoside-Rb_2_	Y=31.68X+246.74	0.9882	3.90~125.00	0.97	3.00	2.23	3.55	7.01
Ginsenoside-Rc	Y=20.13X+25.79	0.9921	3.90~125.00	1.25	3.00	2.02	3.92	4.21
Ginsenoside-Rd	Y=13.60X+69.00	0.9993	3.90~125.00	0.75	1.95	4.18	4.71	3.16
Ginsenoside-Re	Y=18.52X+136.68	0.9973	3.90~125.00	0.48	1.95	3.34	5.06	4.67
Ginsenoside-Rf	Y=37.66X+473.14	0.9952	3.90~125.00	0.97	3.00	2.05	3.12	5.03
Ginsenoside-Rg_1_	Y=52.38X+109.20	0.9994	3.90~125.00	0.48	1.50	2.29	2.49	4.12
Aconitine	Y=6193.52X-80.34	0.9996	0.03~1.25	0.01	0.04	2.76	3.51	4.45
Mesaconitine	Y=3617.22X-63.02	0.9939	0.03~1.25	0.01	0.04	3.97	3.28	4.96
Hypaconitine	Y=1207.82X+180.44	0.9960	0.19~6.24	0.01	0.04	2.55	2.42	4.81

**Table 3:** Analsysis of the recovery of ten compounds in Shen-Fu decoction

**Compounds**	**Initial amount(ng)**	**Added amount(ng)**	**Detected amount(ng)**	**Recovery (%)**	**RSD/% (n=5)**
Ginsenosid-Re	2074.12	1700	3673.54 ± 230.37	94.08	6.27
	2074.12	2100	3991.27 ± 117.73	91.29	2.95
	2074.12	2500	4475.56 ± 236.76	96.06	5.29
Ginsenoside-Rg_1_	2260.51	1800	3959.88 ± 307.37	94.41	7.76
	2260.51	2250	4310.87 ± 229.80	91.13	5.33
	2260.51	2700	5176.34 ± 166.16	107.99	3.21
Ginsenoside-Rb_1_	2423.46	2000	4299.39 ± 196.65	93.80	4.57
	2423.46	2500	4812.75 ±185.92	95.57	3.86
	2423.46	3000	5271.72 ± 130.37	94.94	2.47
Ginsenoside-Rc	2231.62	1800	3933.62 ± 155.38	94.56	3.95
	2231.62	2250	4644.8 ± 231.31	107.25	4.98
	2231.62	2700	5043.67 ± 244.21	104.15	4.84
Ginsenoside-Rb_2_	1597.95	1200	2880.31 ± 83.82	106.86	2.91
	1597.95	1500	3277.51 ± 128.48	111.97	3.92
	1597.95	1800	3510.26 ± 169.97	106.24	4.84
Ginsenoside-Rd	816.73	640	1416.19 ± 86.39	93.67	6.10
	816.73	800	1642.99 ± 95.82	103.28	5.83
	816.73	960	1700.19 ± 80.93	92.03	4.76
Ginsenoside-Rf	2000.12	1600	3563.83 ± 252.68	97.73	7.09
	2000.12	2000	4173.63 ± 261.27	108.68	6.26
	2000.12	2400	4549.1 ± 256.57	106.21	5.64
Aconitine	2.91	2.4	5.52 ± 0.27	108.75	4.89
	2.91	3.0	5.67 ± 0.31	92.00	5.47
	2.91	3.6	6.7 ± 0.34	105.28	5.07
Mesaconitine	7.12	5.6	12.98 ± 0.66	104.64	5.08
	7.12	7.0	13.74 ± 0.68	94.57	4.95
	7.12	8.4	15.82 ± 0.56	103.57	3.54
Hypaconitine	100.06	80	174.69 ± 7.95	93.29	4.55
	100.06	100	204.9 ± 10.90	104.84	5.32
	100.06	120	225.07 ± 7.81	104.18	3.47

### Sample analysis

The described RRLC-QQQ-MS/MS method was subsequently applied to the analysis of Shen-Fu decoction, made by authenticated Radix ginseng and aconite root (see method part). The quantitative analytical results are shown in Table 4. The repeatability of the ten analytes in the Shen-Fu decoction was reliable (RSD<6.28%). From Table 4, Shen-Fu decoction showed higher amounts of ginsenosides than alkaloids. This result meant that the Shen-Fu decoction may have very low toxicity levels, as aconitine, hypacoitine and mesaconitine are the main toxicity source of some toxic herbal medicines [27]. Furthermore, ginsenoside-Rb1 was the most abundant of the ten compounds in Shen-Fu decoction. Conversely, aconitine was shown to be the least abundant of the ten compounds in Shen-Fu decoction. This method would allow for comparison of the quantity of ginsenosides and alkaloids between Shen-Fu decoction preparations and could therefore be used as a rapid and reliable approach for assessment of the quality of Shen-Fu decoction.

**Table 4:** **Contents of ten compounds in Shen**-**Fu decoction**

**Samples**	**Content (μg/g)**									
	**Rb**_**1**_	**Rd**	**Re**	**Rf**	**Rg**_**1**_	**Rc**	**Rb**_**2**_	**Aconitine**	**Mesaconitine**	**Hypaconitine**
	247.17±11.27	84.21±4.31	210.64±12.66	204.66±10.24	231.22±11.75	223.19±14.01	121.16±7.41	0.21±0.01	0.76±0.04	10.05±0.48

## Materials and methods

### Chemicals, standards and samples

HPLC grade acetonitrile was purchased from Merck (Germany) and MS grade formic acid from Sigma-Aldrich. All other chemicals and solvents were of an analytical grade. Ultra-pure water (18.2MΩ) was prepared with a Milli-Q water purification system (Millipore, Bedford, MA, USA).

The standards reference samples of Ginsenosides Rb_1_, Rb_2_, Rc, Rd, Rg_1_, Re, Rf, AC, HA and MA were purchased from the National Institute for Control of Pharmaceutical and Biological Products (Beijing, China). The purity of the standards was relatively high at no less than 98%. Radix ginseng was purchased from Liaoning luyuan Pharmaceutical Co., Ltd. in China. The processed aconite root was purchased from Tong-Ren-Tang Pharmaceutical store (Beijing, PR China). Panax ginseng and the prepared aconite root were authenticated by Professor Xirong, He, Insitute of traditional Chinese medicine, China Academy of Chinese Medical Sciences.

### Sample preparation

#### Reference standards solutions

Stock solutions were prepared by accurate measurement of ginsenoside Re, Rg_1_, Rf, Rb_1_, Rc, Rb_2_, Rd, aconitine, hypacoitine and mesaconitine. They were dissolved with methanol respectively to get ten reference standards stock solutions (1.0 mg mL^-1^), and were stored at 4°C.

#### Extracts of shen-fu decoction

ShenFu Formula (SF) was prepared by combining of Radix ginseng and the processed aconite root (at a ratio of 3:2). Dried and pulverized white ginseng (18 g) and the processed aconite root (12 g) were ground and then refluxed three times with 300 mL of water for 60 min at 100°C. After cooling, the extracted solutions were filtered under vacuum. The solutions were condensed under decompression and finally were freeze-dried. The decoction extract was dissolved in a measured volume of water with a concentration equal to 10 mg of crude botanicals per milliliter. 1mL of the solution was precipitated with 8 mL ethanol allowed to sit for 24 h at 4°C. The solution was filtered under vacuum. The filtrate was transferred to a 50 mL volumetric flask. Prior to injection, all samples were filtered through a 0.22 μm membrane filter.

### RRLC-MS conditions

An Agilent-1200 RRLC/6410A QQQ system (Agilent, MA, USA) equipped with an electrospray ionization (ESI) source and operated in positive ion mode (data analysis software Masshunter version B.01.04) was used for the simultaneous determination of seven ginsenosides and three aconitum alkaloids in Shen-Fu decoction. The separation was performed on an Agilent ZORBAX C18 SB column (100 mm×2.1 mm, 1.8 μm). The gradient mobile phases consisted of (A) water containing 0.05% formic acid and (B) acetonitrile for gradient elution from the column at 40°C. The linear gradient conditions assessed for gradient optimization were as follows: 0–2 min, 28-34% B; 2–6 min, 34-35%; 6–10 min, 35-100%; 10–11 min, 100%. The flow rate was 0.35 ml/min. The column temperature was 40°C. The conditions for MS analysis were as follows: drying gas N_2_ flow rate 12 L min^-1^, gas temperature 350°C and nebulizer pressure was 50 psi. The capillary voltage was set to 4000 V. MRM was employed for quantification. The precursor-to-product ion pair, fragmentor voltage (Frag V) and collision energy (CE) for each analyte are described (Table 1). The dwell time of each ion pair was 200 ms.

### Method validation

An external calibration method was used for quantitative analysis with the linear calibration curves constructed using six different concentrations of the ten compounds. Each concentration was analyzed in triplicate and then the calibration curves were constructed by plotting the peak areas versus the concentrations of each analyte. The LOD and limit of quantification (LOQ) were measured with the signal-to-noise ratios of 3:1 and 10:1, respectively. The intra-day precision was determined by analysis of the standard solution at six times within 1 day. Inter-day precision on other hand, was determined by repeated analysis of the sample for three consecutive days. For the assessment of experimental repeatability test, five independent sample solutions were prepared by the procedures noted in *Extracts of Shen*-*Fu decoction*. The recovery of this method was determined using the standard addition method. Three different concentration levels (approximately equivalent to 0.8, 1.0 and 1.2 times of the concentration of the original amount in the matrix) of the references standards were added into the sample in triplicate. The average recoveries were determined by the following equation: Recovery(*%*) = (Observed amount - Original amount)/Spiked amount × 100*%*, RSD (*%*) = (SD/mean) × 100*%*.

## Conclusions

This is the first report of the simultaneous determination of the major compounds in Shen-Fu decoction. By using RRLC coupled with an ESI triple quadrupole tandem spectrometer, we developed and validated a rapid, simple and reliable method to simultaneously determine ten marker chemicals (ginsenoside Re, Rg_1_, Rb_1_, Rc, Rb_2_, Rd, Rf, aconitine, hypacoitine and mesaconitine) in the Shen-Fu decoction. This method provides an excellent quantitative tool for the quality assessments of TCM formulae because of its high capacity, high sensitivity, high selectivity and short analysis time.

## Abbreviations

RRLC-MS/MS: Rapid resolution liquid chromatography coupled with tandem mass spectrometry; QQQ-MS/MS: Triple quadrupole tandem mass spectrometer; RSD: Relative standard deviations; TCMs: Traditional Chinese medicines; SFI: Shen-Fu Injection; SIM: Selected ion monitor; MRM: Multiple reaction monitor; ESI: Electrospray ionization; Frag V: Fragmentor voltage; CE: Collision energy; LOD: Limit of detection; LOQ: Limit of quantification; NEAs: Nonester alkaloids; MDAs: Monoester diterpene alkaloids; DDAs: Diester diterpene alkaloids; AC: Aconitine; MA: Measaconitine; HA: Hypaconitine.

## Competing interests

The authors declare that they have no competing interests.

## Authors’ contributions

GN, M-GZ and D-DQ conceived of the study, participated in its design and coordination, and drafted the manuscript. GN, L-MT and Y-DW performed experiments and analyzed results and helped to draft the manuscript. HY, N-XH, W-RF and LY helped to do experiments. All authors read and approved the manuscript.
